# Efficient plasma electron accelerator driven by linearly chirped multi-10-TW laser pulses

**DOI:** 10.1038/s41598-023-28755-1

**Published:** 2023-02-20

**Authors:** A. Grigoriadis, G. Andrianaki, I. Tazes, V. Dimitriou, M. Tatarakis, E. P. Benis, N. A. Papadogiannis

**Affiliations:** 1 Institute of Plasma Physics and Lasers, Hellenic Mediterranean University, 74100 Rethymno, Greece; 2 Department of Physics, University of Ioannina, 45110 Ioannina, Greece; 3 School of Production Engineering and Management, Technical University of Crete, Chania, Greece; 4 Department of Electronic Engineering, Hellenic Mediterranean University, 73133 Chania, Greece; 5 Physical Acoustics and Optoacoustics Laboratory, Department of Music Technology and Acoustics, Hellenic Mediterranean University, 74100 Rethymnon, Greece

**Keywords:** Laser-produced plasmas, Plasma-based accelerators, Ultrafast lasers

## Abstract

The temporal rearrangement of the spectral components of an ultrafast and intense laser pulse, i.e., the chirp of the pulse, offers significant possibilities for controlling its interaction with matter and plasma. In the propagation of ultra-strong laser pulses within the self-induced plasma, laser pulse chirp can play a major role in the dynamics of wakefield and plasma bubble formation, as well as in the electron injection and related electron acceleration. Here, we experimentally demonstrate the control of the generation efficiency of a relativistic electron beam, with respect to maximum electron energy and current, by accurately varying the chirp value of a multi-10-TW laser pulse. We explicitly show that positively chirped laser pulses, i.e., pulses with instantaneous frequency increasing with time, accelerate electrons in the order of 100 MeV much more efficiently in comparison to unchirped or negatively chirped pulses. Corresponding Particle-In-Cell simulations strongly support the experimental results, depicting a smoother plasma bubble density distribution and electron injection conditions that favor the maximum acceleration of the electron beam, when positively chirped laser pulses are used. Our results, aside from extending the validity of similar studies reported for PW laser pulses, provide the ground for understanding the subtle dynamics of an efficient plasma electron accelerator driven by chirped laser pulses.

## Introduction

It has been more than 2 decades since the first experimental demonstrations of the generation of relativistic electron beams produced by the interaction of ultra-strong laser pulses with plasma^1–8^. Ever since, several research groups worldwide invested considerable effort towards understanding and detailing the Physics behind the mechanism of Laser Wakefield Acceleration (LWFA), that drives the formation and dynamics of the relativistic electron beams^9–12^. In addition, efforts were directed towards improving the generated electron beam quality characteristics (brightness, maximum energy, quasi-monochromaticity, divergence and stability) in order to harness it as a reliable secondary high energy electron source with potential use in various applications (e.g. radiation dosimetry^13^, generation of anti-matter^14^, and electron diffraction^15^). Finally, these early studies have additionally initiated similar investigations related to the generation of betatron-type X-ray radiation^16–18^, as well as to the Inertial Confinement Fusion (ICF)^19,20^.

The efficiency of the electron acceleration process, considering its aforementioned characteristics, depends on parameters that are related mainly to the target gas density profile as well as to the ultra-strong laser pulse characteristics. These parameters essentially affect the efficiency of the plasma bubble formation and correspondingly the injection mechanisms, as described within the LWFA concept. In the literature there is a number of studies reporting on measurements of relativistic beam electron spectra based on various injection mechanisms such as, self-injection, ionization injection^18,21,22^, down-ramp injection^23,24^ and colliding pulse^25^. Most interestingly, the variation on the laser pulse temporal content, i.e., the chirp of the laser pulse, was used with PW laser pulses to control the generation of relativistic electrons in the GeV energy range^26,27^.

Earlier reports experimentally explored the role of PW-class laser chirp, by varying simultaneously the second and the third order, in the production of electron beams in GeV energies^26,27^. The physical mechanisms for electron acceleration in PW laser intensities are in general different compared to those of multi-10-TW class lasers. In this work, we report on the role of multi-10-TW class laser chirp in the production of electron beams in mutli-10-MeV energies. The main finding of our study is that in few tens of TW laser power, the maximum electron energy can be easily controlled just by varying only the linear part of the laser chirp (second order). Especially, we show that at certain value of positive second order laser chirp the maximum energy of the electrons is extended more than 50%, compared to the Fourier-Transform Limited (FTL) case. This extension of the maximum electron energy has also been reported for PW power lasers and for positively chirped laser pulses, and there it was attributed to the higher ponderomotive potential of the electric field^26,27^. However, our study based on results of Particle-In-Cell (PIC) simulations that fully support our experimental findings, clearly shows that, at least for mutli-10-TW class lasers, this is not the case. In our conditions, higher ponderomotive potentials result in lower efficiency electron trapping inside the bubble due to higher wavebreaking E-field limit. This is also supported by introducing the laser chirp parameter into well-established plasma physics equations. The latter happens for the negatively chirped pulses. On the contrary, for positively chirped pulses, wavebreaking takes place at lower laser electric fields. In this case, few ps after the initiation of the laser-plasma interaction, a very localized electron bunch is formed inside the plasma bubble and accelerated to relatively higher energies.

**Figure 1:**
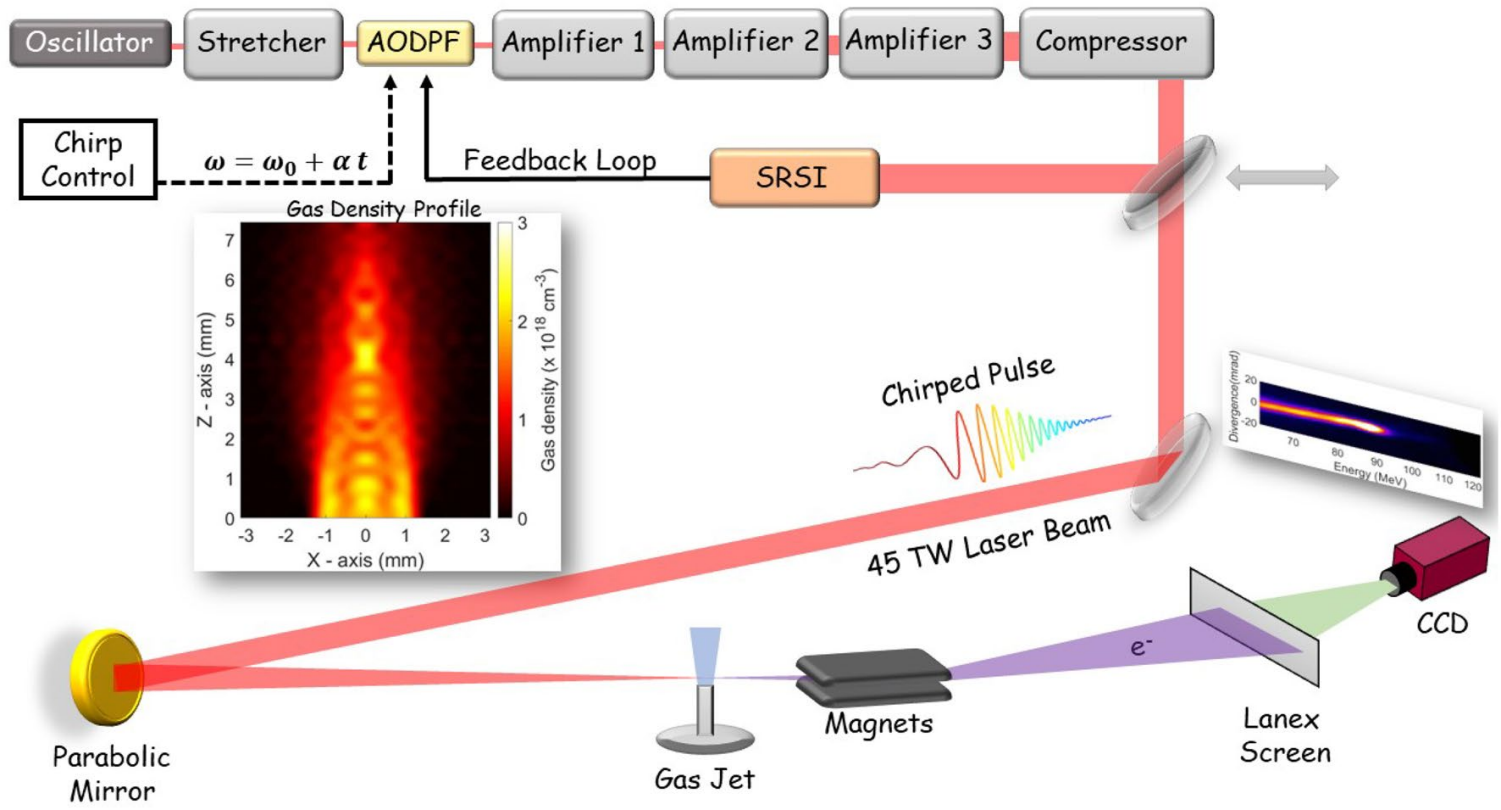
Schematic layout for the generation of MeV electron beams using chirped laser pulses delivered by the “Zeus” 45 TW laser system. The accurate control of the chirp value of the laser pulses is achieved by an acousto-optic dispersive programmable filter (AODPF), located prior to the three amplification stages. The laser pulse duration is measured after the final compression by a self-referenced spectral interferometry (SRSI) autocorrelator, which operates in feedback with the AODPF, to deliver the optimum temporal pulse shape. The laser beam is driven to the interaction chamber, where it is focused onto a pulsed He gas jet by an *f*/18 parabolic mirror. The resulting accelerated electrons are energy analysed by a uniform magnetic field, formed by two parallel-plate magnets, and driven onto a scintillating screen (Lanex Regular). Their spectrum is finally recorded by a CCD camera attached to the scintillating screen. The left inset picture shows the measured gas density profile.

## Results

### Electron spectral images

The experimental setup is shown in Fig. 1 and described in detail, along with the measurement process, in section “Methods”.

**Figure 2:**
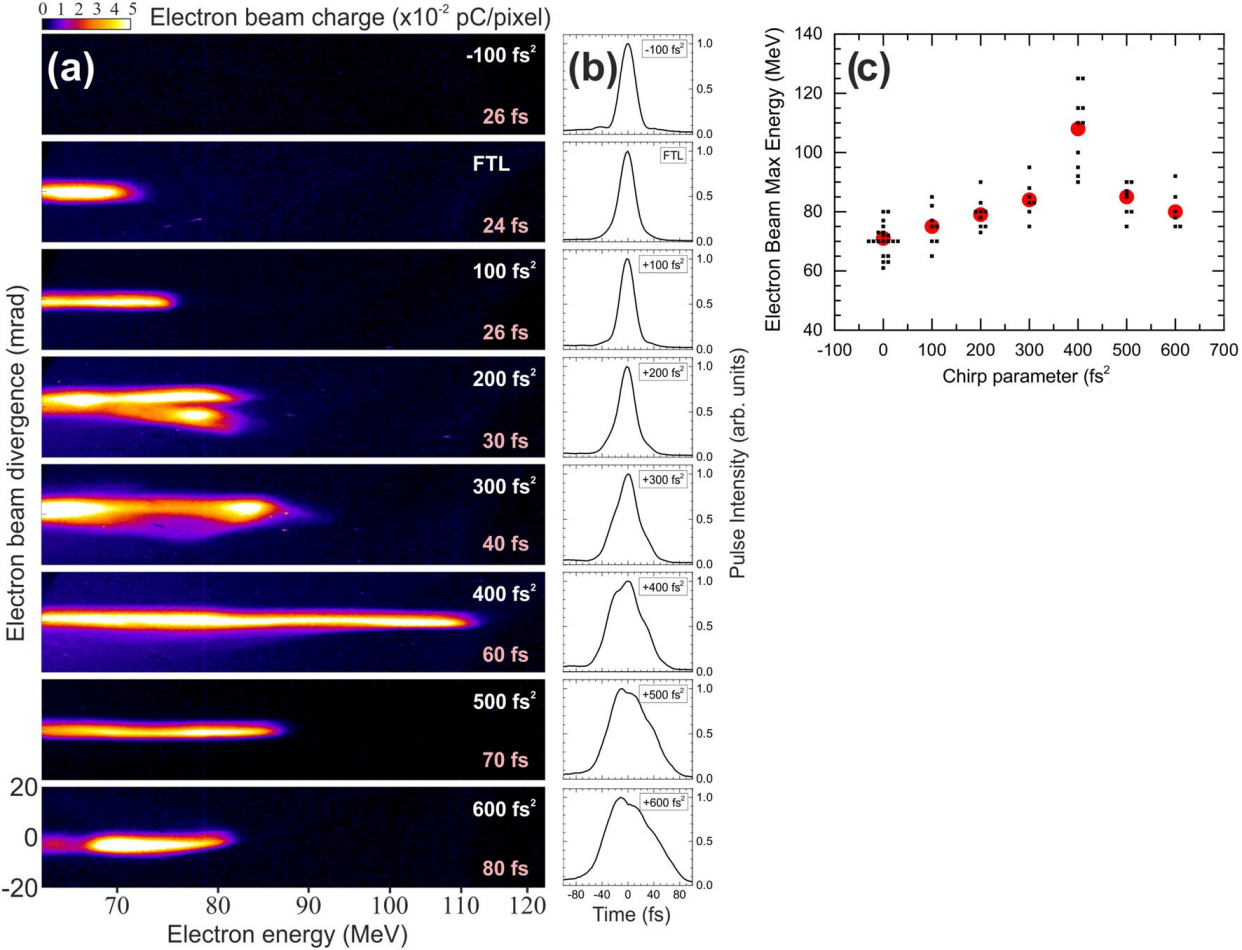
(**a**) Representative measurements of electron spectral images obtained by the interaction of 826 mJ chirped laser pulses with He gas-jet targets. The spectral images cover a kinetic energy range from 62 to 122 MeV, as allowed by the magnetic spectrometer geometry and scintillating screen dimensions. Note that electron spectra for negative chirp values showed no measurable electron signals, a fact that is attributed to their lower than 62 MeV kinetic energies. The spectral image for the $-100$ fs$^2$, shown at the top panel, is typical for all the negatively chirped laser pulses used in this study. (**b**) The corresponding to the measurements of (**a**) FTL and chirped laser pulses intensity temporal shapes. (**c**) Reproducibility measurements of the maximum electron energy (measured at 1/e) for FTL and chirped laser pulses. The black filled squares correspond to the individual measurements. Note that identical maximum energy values have been closely separated in the chirp axis for better visibility. Large red filled circles correspond to the averaged values for each chirp.

In Fig. 2a, we show representative measurements of MeV electron spectral images obtained by the interaction of 826 mJ chirped laser pulses with He targets. For this study we have first optimized the electron acceleration conditions (gas pressure, focusing geometry with respect to the gas jet, pulse jet timing, laser pulse energy) for the FTL case. From Fig. 2a it is evident that the FTL pulses show a relative monochromaticity. Then, in order to investigate the effect of the laser chirp, we kept the optimized for the FTL pulse conditions identical for the rest of the measurements. Starting from a FTL pulse (duration 24 fs measured at FWHM), the laser pulse chirp was accurately varied in a controlled manner in steps of 100 fs$^2$, covering values from $-600$ to $+600$ fs$^2$ (duration 80 fs). The electron spectral images indicate a significant overall enhancement of the maximum electron energy for positive chirp values, as compared to that of the FTL pulse. The maximum electron energy is seen to increase with increasing the positive chirp value up to the maximum energy of 110 MeV, recorded for the case of $+400$ fs$^2$. Higher positive chirp values result in lower maximum electron energies, however, still higher than that of the FTL case. A decrease of the maximum electron energy for large chirp values is expected due to the significant lowering of the laser pulse peak intensity (more than a factor of three for $+600$ fs$^2$). Thus, the decrease above $+400$ fs$^2$ could be attributed to this effect. Surprisingly, no electron spectra were recorded within our energy observation window for all the negative chirp values, as indicated by the top panel in Fig. 2a. Considering that the minimum detection energy threshold of our magnetic spectrometer is 62 MeV, determined by the magnetic spectrometer geometry and scintillating screen dimensions, generated electrons with maximum kinetic energy below this limit are not detected. Thus, the electron beam acceleration with negatively chirped pulses is expected to result in quite lower kinetic energies compared to the positively chirped or FTL pulses. Furthermore, the total integrated electron beam charge, for the kinetic energy widow between 62 and 122 MeV, presents a similar behavior as the maximum kinetic energy. There is an increase with the increase of the chirp value, maximized for the chirp value of $+400$ fs$^2$, having a 3 times higher total charge than that for the FTL. Then, it slowly drops as the chirp value keeps increasing. In Fig. 2b, we also present the corresponding to the measurements of Fig. 2a FTL and chirped laser pulses intensity temporal shapes. The overall symmetric pulse shapes exclude any electron acceleration effects induced by considerable pulses asymmetries. It should be emphasized that for negative chirp values, in a series of measurements, we could not detect electrons within the energy detection window. This was not the case for the FTL and all the positively chirped pulses, where in a series of measurements, in most of the cases we observed electrons within the energy detection window, that had maximum energies quite close to the typical spectra shown in Fig. 2c.

### PIC simulations and theory

In order to shed light to the underlying dynamics responsible for our experimental findings, we performed PIC simulations which are described in section “Methods”. Specifically, we compare PIC simulations for three cases of laser pulses, i.e., a FTL pulse and two symmetric chirped pulses with chirp values of $-400$ fs$^2$ and $+400$ fs$^2$, respectively, where for the latter the maximum electron energy is observed.

**Figure 3:**
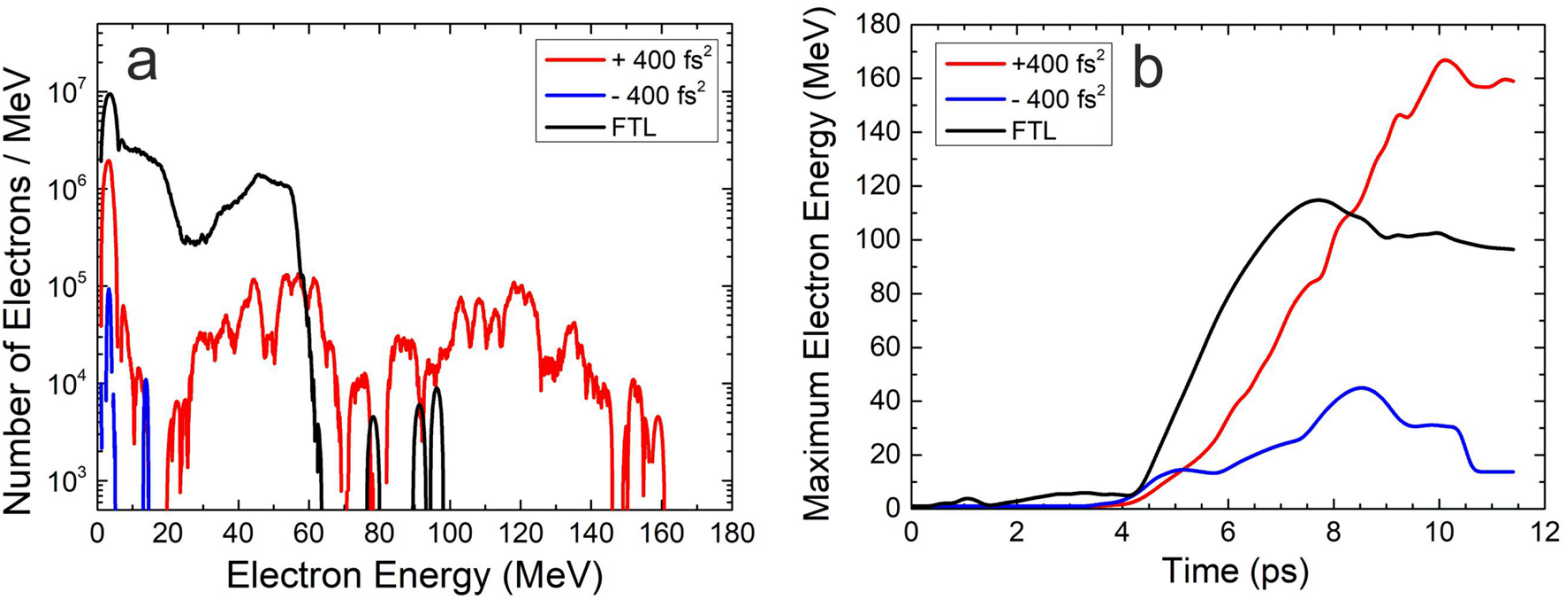
PIC simulation results. (**a**) MeV electron energy spectra for 29 TW laser pulses with positive chirp ($+400$ fs$^2$, red solid line), negative chirp ($-400$ fs$^2$, blue solid line), and no chirp (FTL, black solid line), interacting with He gas-jet targets. Positively chirped pulses are seen to generate the highest maximum electron energy compared to the negatively chirped and the FTL pulses. This behavior is in qualitative agreement with corresponding experimental results presented in Fig. 2a. (**b**) Temporal evolution of the maximum electron energy for the chirped and FTL laser pulses. Positively chirped pulses show a large energy gain rate that is wider in time, compared to the negatively chirped and FTL pulses, thus resulting in the highest maximum electron energy.

In Fig. 3a, we present the generated electron spectra for the above cases. It is clearly seen that PIC simulations qualitatively reproduce the experimental findings. They predict a higher maximum electron energy for the positively chirped pulses than for the FTL pulses, while for the negatively chirped pulses the electron production is limited to much lower kinetic energies, that lie outside of our experimental detection limits. A dynamic picture of the above results is given in Fig. 3b, where the temporal evolution of the maximum electron energy over the duration of the simulation is presented. There, it is evident that the electron acceleration starts at the same time of $\simeq 4$ ps for all three cases. Positively chirped pulses are seen to have a large energy gain rate that lasts for $\simeq 6$ ps, and then reach the final maximum value. FTL pulses have an even larger energy gain rate, which however lasts for only $\simeq 3.5$ ps, followed by a $\simeq 20\%$ reduction compared to the maximum value. This indicates that electron beam suffers an appreciable deceleration during the last $\simeq 50\%$ of its temporal evolution. As a result, positively chirped pulses end up with a higher maximum electron energy than FTL pulses. Negatively chirped pulses show the smallest energy gain rate that lasts for $\simeq 4.5$ ps, followed by a $\simeq 60\%$ reduction. Thus, the electron beam suffers a severe deceleration after $\simeq 40\%$ of its temporal evolution, resulting to the lowest maximum electron energy, as compared to both FTL and positively chirped pulses.

For a deeper understanding of our findings, we proceeded to examine the dynamics of the LWFA mechanism. In the LWFA picture, the laser pulse propagates through the ionized medium with a group velocity, $u_g$^28^

1 $$\begin{aligned} \frac{u_g}{c} = 1 - \frac{3}{2} \frac{\omega ^2_p}{\omega _0^2}, \end{aligned}$$

where $\omega _0$ and $\omega _p$, are the laser and plasma frequencies, respectively. In order to account for the chirp of the laser pulses we modified Eq.  (1) as:

2 $$\begin{aligned} \frac{u_g}{c} = 1 - \frac{3}{2} \frac{\omega ^2_p}{[ \omega _0 \pm a (t - t_0)]^2}, \end{aligned}$$

where *a* is the chirp parameter and $t_0$ the time corresponding to the peak of the pulse. Note that the chirp values, given in units of fs$^2$, correspond to the reciprocal of the chirp parameter, i.e., 1/*a*. Equation (2) indicates that the group velocity at the leading edge of the laser pulse ($t<t_0$), which is roughly equal to the bubble propagation velocity^28^, is lower for a positively chirped pulse compared to a negatively chirped one^29^. It has been reported^30–32^ that the maximum electric field limit, before the electron density wavebreaking occurs, is directly associated to the electron density velocity. For a cold relativistic plasma the maximum electric field amplitude that can be sustained before the wavebreaking is^30^:

3 $$\begin{aligned} E_{max} = \frac{m_e c \omega _p}{e} \sqrt{2(\gamma - 1)}, \end{aligned}$$

where $m_e$ and *e* are the electron mass and charge, respectively, and $\gamma =(1-(u/c)^2)^{-1}$ is the relativistic Lorentz factor. Thus, positively chirped pulses, which have smaller group velocities than the negatively chirped pulses, are expected to allow for a wavebreaking at lower electric field amplitude values compared to the ones corresponding to the negatively chirped pulses. However, the high plasma electric field value is not the only necessary condition that should be fulfilled to efficiently accelerate electrons in higher energies. The conditions for the efficient acceleration of electrons as a function of the laser chirp are investigated with PIC simulations below.

**Figure 4:**
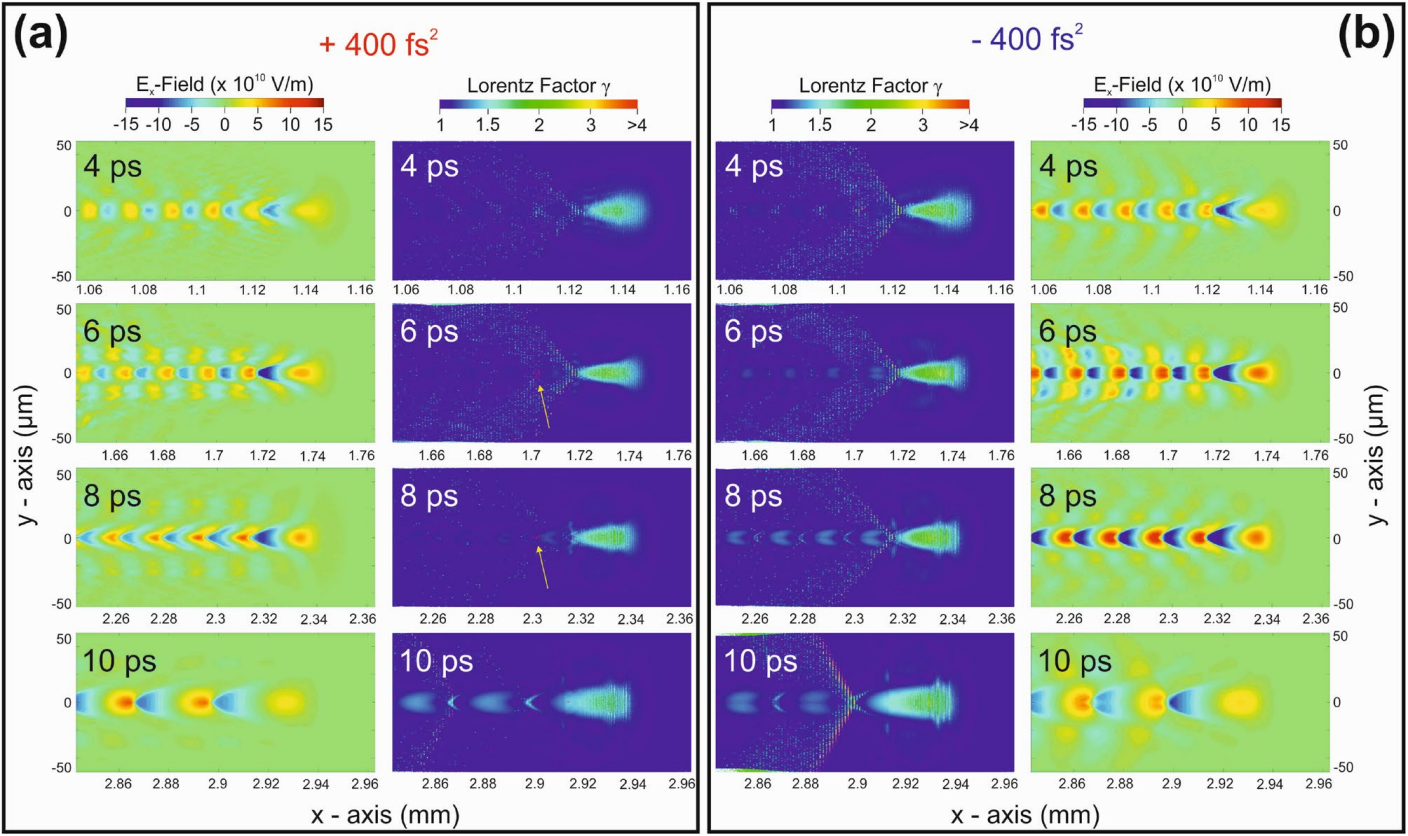
PIC simulation results. (**a**) Spatial distribution of the plasma electric field (left) and the relativistic Lorentz factor, $\gamma$, (right) for a chirped laser pulse with chirp value of $+400$ fs$^2$, obtained at 4, 6, 8, and 10 ps after the simulation was initiated (see text for details). The yellow arrows depict the large $\gamma$ values at the rear end of the bubble. (**b**) Same as in (**a**) but for a chirped laser pulse with chirp value of $-400$ fs$^2$.

Supporting evidence about this prediction can be obtained by comparing the PIC simulation results for the spatial distribution of the relativistic Lorentz factor, $\gamma$, for two laser pulses of equal duration and peak intensity, i.e., a chirped laser pulse with symmetric chirp values of $+400$ fs$^2$ and $-400$ fs$^2$, respectively. Such a comparison is presented in Fig. 4 for the evolution times of 4, 6, 8, and 10 ps, along with the corresponding plasma electric field distributions. A direct comparison between the negative and positive cases at 4 ps, i.e., where the process of electron acceleration initiates, shows that for the negatively chirped pulses the plasma electrons experience a steeper and stronger force at the forefront of the laser pulse as well as at the plasma bubble walls. This is in accordance with the corresponding plasma electric field distributions also shown in Fig. 4. As a result, a spatially broader electron acceleration distribution is formed that does not favor the guidance of the electrons at the rear end of the bubble to initiate the self-injection mechanism. The acceleration picture becomes clearer at 6 ps. For the positively chirped pulses the electrons are driven to the rear end of the bubble and thus are susceptible to self-injection and subsequent acceleration to maximum allowed kinetic energy values. On the contrary, for the negatively chirped pulses, the electrons experience wide spatial distributions that may result in injection at the broader rear end of the bubble. This effect will result to smaller maximum kinetic energies as compared to the corresponding positive chirp case. Later, at 8 ps, where the acceleration process is peaking, as shown in Fig. 3, it is clearly seen that for the positively chirped pulses the electrons are mostly distributed at the rear end of the bubble, thus augmenting the self-injection process and the maximum attained kinetic energy. At the same time, the negatively chirped pulses are seen to fill the bubble with electrons having a broad spatial distribution, that cannot account for an acceleration at maximum attainable kinetic energies. Finally, at 10 ps, close to the end of the acceleration process, it is seen that, for the positive chirp case, the electron beam injected at the rear end of the bubble at earlier times reaches the front end of the bubble, thus gaining the maximum attainable kinetic energy. On the contrary, for the case of negative chirp, electrons are seen to be distributed all over the volume of the bubble, while at the same time they strongly depart from the formation of the second bubble in row. As a general remark, we may say that, for the negatively chirped pulses, electrons are seen to propagate to the back of the bubble formation at higher $\gamma$ values, and thus at higher velocities compared to the positively chirped pulse, in agreement to the predictions of Eq. (3).

From the above results, it becomes clear that the introduction of linear chirp in a laser pulse has direct and significant effects on the generation of relativistic electron beams, owed intrinsically to the bubble formation geometry and the related electron injection and acceleration mechanism. We further investigated with PIC simulations this behavior by monitoring the spatial evolution of the plasma density bubble formation for the three aforementioned cases of laser pulses (FTL, $+400$ fs$^2$, and $-400$ fs$^2$).

**Figure 5:**
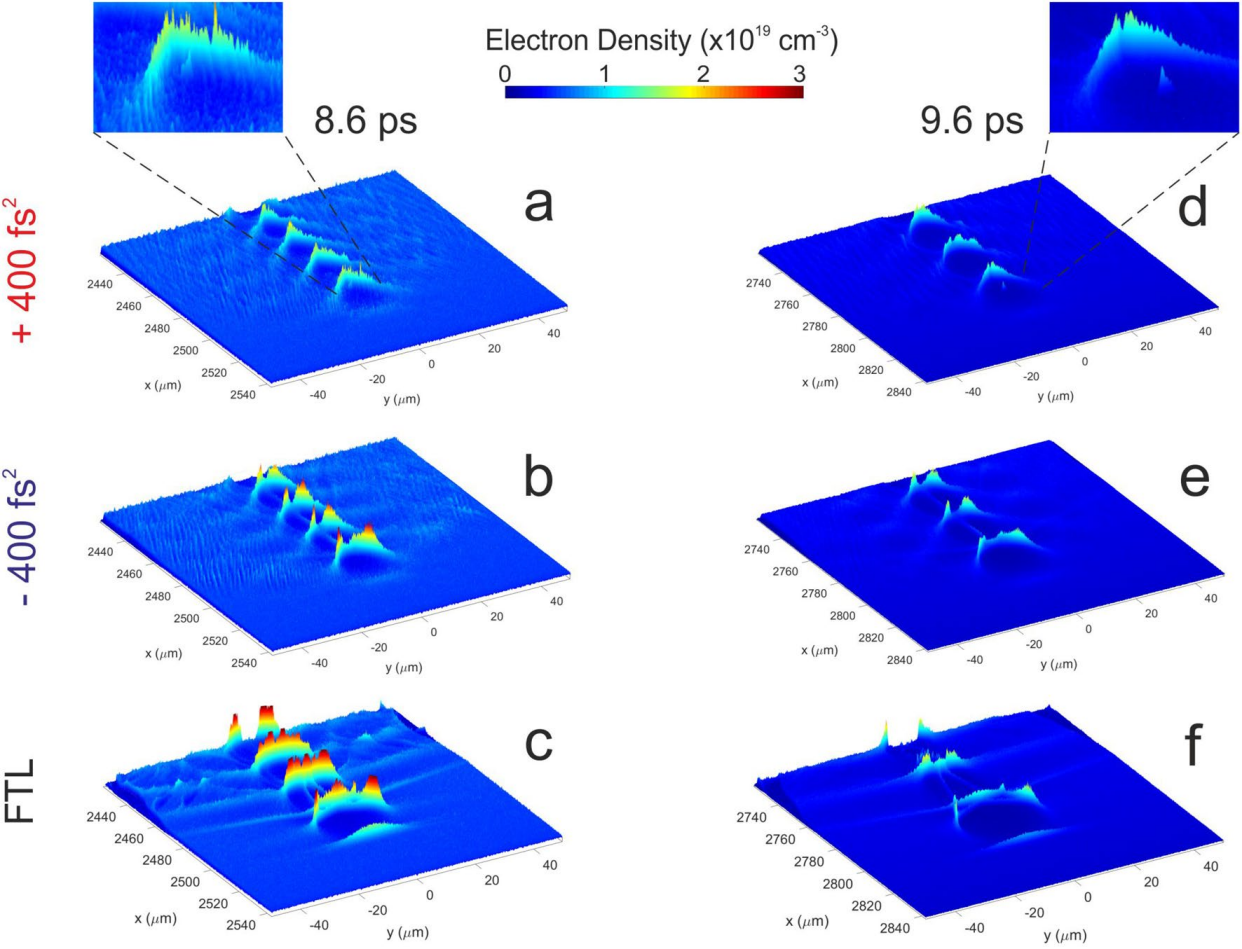
PIC simulation results. Formation of bubble plasma density for FTL pulses as well as positively ($+400$ fs$^2$) and negatively ($-400$ fs$^2$) chirped pulses at PIC evolution times of 8.6 ps (**a**–**c**) and 9.6 ps (**d**–**f**). A smoother plasma density bubble is evident for the positively chirped pulses that promotes the wavebreaking and subsequent acceleration of the electron beam. The wavebreaking and acceleration of the self-injected electrons for the case of $+400$ fs$^2$ chirped pulses are clearly depicted in the zoomed pictures of graphs (**a**,**d**).

In Fig. 5, we present the simulated bubble formation for the three chirp cases for the evolution times of 8.6 ps and 9.6 ps, where the wavebreaking picture is most clearly seen. At 8.6 ps, a well-formed bubble with a smooth density increase peaking at its rear end is evident for the positively chirped pulses. The negatively chirped pulses, as well as the FTL pulses, show a similar electron density increase towards the rear end of the bubble, which however, fluctuates considerably. Moreover, the bubble dimension of the latter is seen to be wider and more elliptic perpendicular to the propagation axis than the one in the positively chirped pulses. These qualitative differences in the geometrical and local density characteristics may cause a wavebreaking in the wider rear area of the bubble, thus resulting in electrons with smaller maximum kinetic energy. It is instructive to see in Fig. 5a–c, that for the positively chirped pulses, the electron wavebraking has been initiated at the most rear part of the first bubble, while in the other cases no visible evidence for an initiation of a strong wavebreaking is seen.

This behavior is repeated in Fig. 5d–f for the PIC evolution time of 9.6 ps, where the density of bubble is reduced, and the electron acceleration is clearer. Again, the acceleration of the electron beam, after the wavebreaking at $\sim$ 8.6 ps, is clearly seen for the positively chirped pulses while it is not graphically visible for the FTL and negatively chirped pulses. This behavior is not only in qualitative agreement with our experimental findings but also exposes the bubble formation dynamics and the underlying injection conditions that control the electron beam acceleration within the LWFA picture.

## Discussion

In this work, we have investigated both experimentally and with PIC simulations the dynamics of plasma bubble formation and the subsequent relativistic electron beam generation with the use of 29 TW chirped laser pulses. The primary goal of our study was to expose the electron acceleration process in conditions of laser pulses with intrinsic spectral temporal rearrangement, in order to seek ways to efficiently control the maximum kinetic energy and current of the electron beam. Such a control is an indispensable characteristic for electron beams serving as secondary relativistic electron sources. Our experimental results clearly show that this goal can be achieved for multi-10-TW power lasers, currently existing as table-top systems in a number of laboratories worldwide. Thus, our results are expected to be of particular interest to the wider community of ultra-strong laser-matter interactions.

Specifically, we found an overall increase of the maximum energy of the electron beam, along with a corresponding increase of the total current, when using positively chirped pulses. The higher maximum energy was achieved for the chirp value of $+400$ fs$^2$, while its value smoothly decreases on either side. It should be emphasized that our experimental findings are in qualitative agreement with similar reports in the literature concerning PW laser pulses and GeV electron beams^26,27^, which however lack a detailed and thorough theoretical justification. Here, we provide a clarified picture of the dynamics of the electron acceleration process involving chirped laser pulses, based on PIC simulations corresponding to our experimental conditions. Thus, our PIC simulations not only qualitatively reproduce the measured electron spectra, but also provide valuable information about the dynamics in the bubble formation and the related wavebreaking mechanism, that largely drives the outcome of the electron acceleration process.

The main finding of our PIC simulations, is that positively chirped pulses result in the evolution of a smooth density distribution of the plasma bubble, peaking at the rear end of the bubble. Negatively chirped and FTL pulses result in wider density distribution geometries with appreciable fluctuations. These results on the dynamic bubble formation are accompanied by the corresponding PIC results on the Lorentz $\gamma$ factor spatial distributions. There, it was clearly shown that for negatively chirped pulses, plasma electrons experience steeper and stronger forces at the forefront of the laser pulse as well as at the plasma bubble walls, thus resulting in a spatially broader electron acceleration distribution. On the other hand, for positively chirped pulses, electrons experience smoother forces, while electrons are seen to be accelerated more efficiently to the most rear end of the bubble. These dynamic pictures strongly affect the wavebreaking and the related electron injection conditions. Wavebreaking at the most rear end of the bubble results in longer acceleration paths, as it happens for the positively chirped pulses. Wavebreaking for FTL and negatively chirped pulses is seen to be concluded within the wider area of the rear end of the bubble, thus resulting in shorter acceleration paths and consequently smaller electron energies. Clearly, our PIC simulations qualitatively reproduce the experimental findings and thus, the dynamical pictures of plasma bubble evolution and related electron acceleration provide new insights in the concept of interaction of multi-10-TW chirped laser pulses with plasma.

## Methods

### Experimental setup

The experiments were performed using the 45 TW fs laser system “Zeus” (Amplitude Technologies) at the Institute of Plasma Physics and Lasers (IPPL) of the Hellenic Mediterranean University^33,34^. The main laser beam has a maximum energy of 1.3 J per pulse, central wavelength at 807 nm and a FTL pulse duration of 24 fs, at a repetition rate up to 10 Hz. The optimisation of the temporal shape of the pulse is performed by an acousto-optic dispersive programmable filter (AODPF, Fastlite Dazzler) located between the temporal stretching at the exit of the oscillator and the three amplification stages (see Fig. 1). AODPF operates in feedback with the self-referenced spectral interferometry (SRSI) autocorrelator (Fastlite Wizzler), located at the exit of the compressor. The beam is fed to the SRSI by a movable mirror to deliver the information to the AODPF filter, operating in a feedback loop to temporally optimize the delivered pulse. Furthermore, the laser operating system allows for a manual modification of the laser pulse temporal structure, by controlling the chirp value of the laser pulse. This is done by accurately varying the second order term *a* of the laser frequency $\omega = \omega _0 \ t+at^2$.

After setting the chirp value and optimizing the laser pulse, the beam was propagated to the experimental chamber and focused on a He gas jet target by a 1 m focal length parabolic mirror (*f*/18). The focal spot at the gas target area was measured to be $30~\upmu$m in diameter (FWHM), and thus, considering a measured at the target pulse energy of 826 mJ, a peak intensity of $(1.05 \pm 0.02)\times 10^{19}$ W/cm$^2$ for the FTL pulses is reached, which corresponds to a normalized vector potential amplitude value of $a_0 = 2.2$. The pulsed He gas jet was automatically controlled, driven by the laser’s repetition rate, by an electromagnetic valve (Parker Valve) having a cylindrical opening of 1 mm diameter. A home-made conical nozzle having a 3 mm exit diameter was adjusted to the valve’s opening. The density profile of the gas jet, for a backing pressure of 35 bar, has been characterized using a Nomarski-type interferometer^35^ and it is presented in the left inset of Fig. 1. Accordingly, since we used helium gas targets that are fully ionized by the pedestal of the laser pulses, the plasma density profile, prior to the interaction with the main laser pulses, is identical with the gas density profile, multiplied in magnitude by a factor of two to account for the two electrons of the ionized helium.

The spectra of the generated relativistic electrons were obtained using a magnetic spectrometer to deflect the electron beam and a combination of a scintillating screen and a charge-coupled device (CCD) camera, as shown in Fig. 1. The magnetic spectrometer consisted of two permanent rectangular magnets (11 cm $\times$ 9 cm) placed in parallel at a separation distance of 1 cm, that resulted in nearly homogeneous magnetic field which was measured with a Hall probe to be 0.64 T. The deflected electron beam was then impinged onto a scintillating screen (Lanex Regular) and the emitted light from the rear side of the screen was imaged by a lens onto the CCD camera. The main laser beam light was blocked by covering the front side of the scintillating screen with a $13~\upmu$m thick Al foil. The electron spectral images were obtained from the recorded CCD images based on the tracking of the relativistic electron trajectories through the magnetic field geometry. Moreover, by calibrating the screen imaging system using a laser light source, we were able to estimate the number of electrons for each electron spectrum^35^.

### Model and PIC simulations

A two-dimensional PIC model was developed and simulated using the EPOCH PIC code^36^ to investigate the experimental findings. Convergence tests were initially performed, as typically done in similar PIC simulations^37^, for variable domain sizes. Specifically, for the direction perpendicular to the laser beam propagation axis, the numerical errors due to reflections of the electromagnetic fields on the boundaries of the domain, were avoided. Additionally, for the direction along the laser beam propagation axis, these simulations helped us eliminate the possibility of data loss due to the computational window size limitations. In this manner, the computational domain size was determined to be $120 \times 100~\upmu$m, with the longer dimension corresponding to the laser beam propagation axis. The domain was discretised by 2400 cells along the laser beam propagation axis and 500 cells perpendicular to it, thus allowing for a higher resolution along the electron beam propagation axis. The number of macro-particles was set to 5 for each cell, using a 3rd order particle shape function. In order to follow the temporal evolution of the plasma bubble, electron injection and subsequent electron acceleration dynamics, a moving window configuration was employed for a simulation time of 11.4 ps.

The laser pulse parameters were set according to the “Zeus” laser pulse characteristics used in the experiments. Thus, Gaussian type laser pulses with maximum intensity, determined by the experimental focusing conditions, and central wavelength of 807 nm were utilized. For the cases of chirped laser pulses, the instantaneous angular frequency, $\omega = \omega _0 \ t+at^2$, was introduced, as it is described in the subsection “Experimental setup”. The PIC simulations ran for three cases of laser pulses: a FTL pulse and two symmetric chirped pulses with chirp values of $1/a=-400$ fs$^2$ and $1/a=+400$ fs$^2$, respectively. These cases correspond to laser pulse durations of 24 fs for the FTL pulse and 60 fs for both the chirped laser pulses. The gas density profile geometry for the He gas jet target in use was obtained from measurements based on interferometric techniques^35^. Thus, considering that the He gas target is a fully ionised by the laser pulse pedestal, the plasma density profile was approximated by a super-Gaussian function, with a maximum density of $4 \times 10^{18}$ cm$^{-3}$.

The PIC simulation setup was proven adequate to support multiple plasma bubble formation and came at affordable computation time using the HPC for Advanced Research Information System (ARIS) of the Greek National Infrastructures for Research and Technology (GRNET)^38^.

## Data Availability

The datasets used and/or analyzed during the current study available from the corresponding author on reasonable request.
